# In Vitro Modeling of the Blood–Brain Barrier for the Study of Physiological Conditions and Alzheimer’s Disease

**DOI:** 10.3390/biom12081136

**Published:** 2022-08-18

**Authors:** Thomas Gabriel Schreiner, Ioana Creangă-Murariu, Bogdan Ionel Tamba, Nicolae Lucanu, Bogdan Ovidiu Popescu

**Affiliations:** 1 Faculty of Medicine, “Carol Davila” University of Medicine and Pharmacy, 050474 Bucharest, Romania; 2 Department of Neurology, “Grigore T. Popa” University of Medicine and Pharmacy, 700115 Iasi, Romania; 3 Department of Electrical Measurements and Materials, Faculty of Electrical Engineering and Information Technology, Gheorghe Asachi Technical University of Iasi, 21-23 Professor Dimitrie Mangeron Blvd., 700050 Iasi, Romania; 4 Advanced Research and Development Center for Experimental Medicine (CEMEX), “Grigore T. Popa” University of Medicine and Pharmacy, Universitatii Str., No. 16, 700155 Iasi, Romania; 5 Department of Applied Electronics and Intelligent Systems, Faculty of Electronics, Telecommunications and Information Technology, Gheorghe Asachi Technical University of Iasi, 21-23 Professor Dimitrie Mangeron Blvd., 700050 Iasi, Romania; 6 Neurology Department, Colentina Clinical Hospital, 020125 Bucharest, Romania; 7 Laboratory of Cell Biology, Neurosciences and Experimental Myology, “Victor Babes” National Institute of Pathology, 050096 Bucharest, Romania

**Keywords:** blood–brain barrier, neurodegeneration, Alzheimer’s disease, in vitro model, organ-on-a-chip, spheroid, human pluripotent stem cells

## Abstract

The blood–brain barrier (BBB) is an essential structure for the maintenance of brain homeostasis. Alterations to the BBB are linked with a myriad of pathological conditions and play a significant role in the onset and evolution of neurodegenerative diseases, including Alzheimer’s disease. Thus, a deeper understanding of the BBB’s structure and function is mandatory for a better knowledge of neurodegenerative disorders and the development of effective therapies. Because studying the BBB in vivo imposes overwhelming difficulties, the in vitro approach remains the main possible way of research. With many in vitro BBB models having been developed over the last years, the main aim of this review is to systematically present the most relevant designs used in neurological research. In the first part of the article, the physiological and structural–functional parameters of the human BBB are detailed. Subsequently, available BBB models are presented in a comparative approach, highlighting their advantages and limitations. Finally, the new perspectives related to the study of Alzheimer’s disease with the help of novel devices that mimic the in vivo human BBB milieu gives the paper significant originality.

## 1. Introduction

The blood–brain barrier (BBB), a unique structure in the human body, is one of the three main barriers along with the blood–leptomeningeal barrier, and the blood–cerebrospinal fluid barrier that separates the brain from peripheral tissues [1]. Its complex cytoarchitecture provides a highly selective environment that allows a bidirectional but strictly controlled passage of different solutes between the brain and the systemic circulation [2]. The structural integrity of the BBB is an essential factor in normal conditions, as BBB’s main role is to protect the sensitive cerebral parenchyma from potential external neurotoxins [3]. Thus, any alteration in the BBB complex leads to reversible and (mostly) irreversible pathological changes in the brain, including neuroinflammation and neurodegeneration [4].

The topic of neurodegenerative diseases has been extensively discussed in recent decades, with extensive research conducted on Alzheimer’s disease (AD) [5,6,7,8]. AD is the most frequent cause of dementia worldwide, with incidence and prevalence expected to rise over the next few years in the context of population aging [9]. AD also possesses a significant socioeconomic burden, with health services dedicated to dementia patients occupying an increasing percentage of the funds allocated to the healthcare system [10]. AD has a definite negative impact on the patients’ quality of life and also indirectly affects society and the patient’s families [11]. Numerous hypotheses have tried to explain AD etiology, the broadly accepted ones suggesting that AD is a result of either the pathological accumulation of amyloid-beta (Aβ) in the brain [12] or the aggregation of other misfolded proteins such as Tau protein [13]. The consequences of complex gene–environmental interaction were also discussed, with viral agents such as human herpesvirus 6 (HHV-6) playing an essential role [14], and finally the outcome of dysregulated mechanisms such as neuroinflammation which switch from their physiological protective functions to a destructive behavior [15]. Despite the extensive experimental data available, no curative treatment currently exists, while there are still many unknowns and incompletely explored pathways.

BBB damage is a common element encountered in AD, among many other neurological disorders including stroke [16], multiple sclerosis [17], and traumatic brain injury [18]. Associated with cerebral dysfunction, BBB permeability dysfunction is an interesting study topic necessary to better understand AD pathophysiology and to find an efficient therapeutic approach. However, the main limitation of studying the human BBB is related to the difficulties encountered when trying to directly assess BBB’s characteristics under in vivo conditions. Because of troublesome accessibility to human BBB, in vitro replication is the only feasible method for direct evaluation.

In the context of growing research conducted on in vitro models of the human BBB, this article aims to offer a comprehensive review of the most significant models used nowadays. After reviewing the physiological structure of the BBB in the first part, the authors offer detailed insights on the already-used BBB models, highlighting their pros and cons. Finally, BBB models that simulate neurodegeneration are suggested for an appropriate in-depth study of AD.

## 2. The Structure and Function of the Blood–Brain Barrier in Physiological Conditions

The BBB’s main role in healthy humans is to ensure brain functioning in physiological parameters. This is achieved via multiple mechanisms that must work in unison continuously and regulate the bidirectional substance between blood and brain parenchyma in a highly selective manner [19]. Firstly, the BBB is a limiting boundary for the potential neurotoxic compounds found in the peripheral circulation. Not only are harmful substances prevented from reaching the brain, but even macromolecules, because of their size and polarity [20]. Additionally, the BBB maintains ion homeostasis, which is essential for the correct functioning of neuronal circuits. Via the Na-K pump and ion channels, the concentration of Na, K, Ca, Mg, and Cl in the central nervous system (CNS) compartment is kept within strict limits, ensuring adequate neuronal functioning and metabolism [21]. Active and passive transport through the BBB is another topic of interest, with the brain microvascular endothelial cells (BMECs) possessing several carriers and specific transporters for hormones and other physiologically active molecules [22]. The regulation of the level of neurotransmitters is also essential for correct neuronal functioning, as an imbalanced level of neurotransmitters such as glutamate can be neurotoxic [23]. Finally, the BBB is crucial in CNS excretion, facilitating the elimination of toxic and residual metabolic end-products [24].

**Figure 1:**
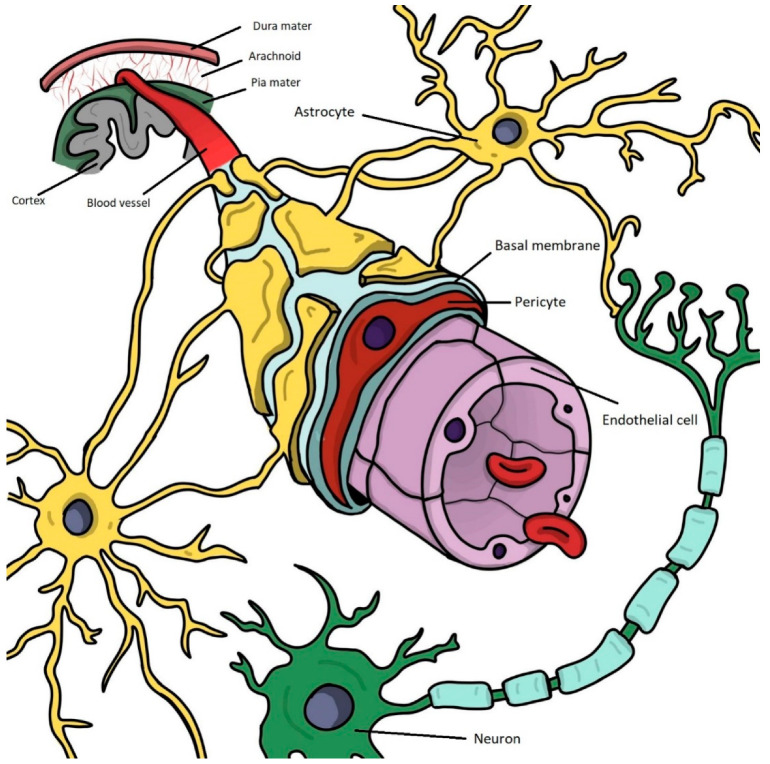
The complex structure of the neurovascular unit in physiological conditions: all components interact anatomically and chemically in a complex web to maintain its functions. Endothelial cells (purple), which make up the main part of the BBB, are characterized by high selectivity in transcellular transport, due to the tight junctions that fuse them together and restrict diffusion across the blood vessels. Pericytes (red) are essential cells in maintaining the structural and functional properties of the BBB and share a common basement membrane (blue) with endothelial cells. Astrocytes (yellow) are involved in supportive processes and have a strategic localization between neurons (green) and other components of the BBB, with their specialized end feet extending to the walls of the blood vessels. (Magda Pîrțac designed this figure by using Adobe Fresco).

To fulfill its physiological functions, the BBB has a complex structure, with the symbiotic involvement of multiple cellular and non-cellular components. While the BBB was considered to be formed by the BMECs, pericytes, and astrocytes in the past, the paradigm has changed during the last decade [25]. As neurons, oligodendrocytes, and microglial cells were also demonstrated to interact with the other abovementioned cells in creating an optimal BBB microenvironment, a relatively new concept is currently used, namely the neurovascular unit (NVU) [26]. Figure 1 highlights the structural complexity of the NVU in normal (health) conditions. As in vitro models of the human BBB, the main focus of this review, have not reached the complexity of including all cellular components encountered in vivo, for the sake of consistency, the term BBB was used throughout the majority of the manuscript. Data on the NVU were also included, as the influence of neurons, oligodendrocytes, and microglia remains essential in both physiological and neurodegenerative conditions; however, the limitations of current in vitro models do not allow the study of these components to reach their full potential.

### 2.1. Brain Microvascular Endothelial Cells

The main component of the BBB is represented by BMECs, a special and unique type of endothelial cells found only at the level of the brain microvessels [27]. Compared to endothelial tissue located in other regions of the human body, the brain endothelium is characterized by a higher selectivity, one of the most important features of the BBB in brain protection [28]. The most relevant particularities of this highly specialized endothelium are the lack of fenestration, the reduced transcellular transport via minimal pinocytosis, and the complex intercellular connections [29]. Indeed, the almost absent paracellular diffusion is a result of the presence of tight junctions (TJs) and adherens junctions (AJs) that connect two neighboring BMECs [30].

The molecular complex of the TJ consists of several proteins, with occludin, claudin, and the junctional adhesion molecule (JAM) being the most relevant ones. Occludin was the first protein identified as a component of the TJ and plays an essential role in stabilizing and regulating the junctional complex [31]. Occludin’s presence in other biologically significant regions (bone, breast, and skin) suggest multiple roles for this protein [32]. Several studies have already shown the association of dysfunctional and/or downregulated occludin to several tumoral pathologies, such as human lung carcinoma [33] and breast cancer metastases [34]. The claudin family (claudin 5, 11, and 12), found at the TJ level, is equally important for structural stability, but also involved in other processes, being linked to the metastasis process and cancer evolution [35]. Finally, JAMs are the third class of functionally diverse proteins involved in several relevant processes in the human body, from modulating cell migration [36] to conferring anatomical stability such as in the case of natural barriers [37].

The transmembrane proteins interact with the actin cytoskeleton of the BMECs via the help of another group of essential scaffolding proteins, the zonula occludens (ZO) proteins (ZO-1, ZO-2, and ZO-3). Moreover, toward the basal part of the cell, at the adherent junction level, transmembrane proteins tighten the paracellular space by binding to the platelet endothelial cell adhesion molecule (PECAM) and cadherin-5 [38]. All these protein components ensure a tight intercellular space and subsequently an impermeable BBB.

BMECs are equally important when studying transcellular substance transport. The myriad of carriers and channels located in both luminal and abluminal sides demonstrates a high degree of selectivity for molecular passage through the BBB. For example, the ATP-binding cassette (ABC) transporter superfamily facilitates the transportation of various substrates against the concentration gradient [39], while the permeability glycoprotein (P-gp) and the multidrug resistance protein (MRP) transporters consisting of other relevant efflux carriers located mostly on the luminal side of the endothelium [40]. These aspects must be taken into consideration when reproducing the human BBB in vitro conditions, especially for drug permeability tests.

### 2.2. Brain Pericytes

Closely connected to the BMECs, pericytes are essential cells in maintaining the structural and functional properties of the BBB. Pericytes are of different subtypes (ensheathing, mesh, and thin-strand) and perform different functions at the CNS level. Firstly, they regulate the cerebral blood flow in different brain regions depending on variations in neuronal activity [41]. The existence of intracellular contractile proteins such as alpha-smooth muscle actin (α-SMA), myosin, and tropomyosin make pericytes capable of contractions [42]. Whether all pericytes have contractile ability remains debatable, as studies demonstrated that only arteriolar pericytes are involved in the hydrodynamic regulation [43].

The support of pericytes for BMECs is manifest from the developmental stages of the cerebral microvasculature, with recent research demonstrating the role of pericytes in the maintenance of a proper brain microcirculation [44]. The loss of pericytes was correlated with dysfunctional cerebral circulation, low blood flow, and subsequent BBB dysfunction with the accumulation of neurotoxins [45]. The pericyte–BMEC intercellular crosstalk is mediated by several growth factors such as angiopoietin 1 (Ang1), transforming growth factor beta (TGF-β), and platelet-derived growth factor-BB (PDGF-BB), which are the main regulators of cell migration at the CNS vascular level [46]. BMEC modulation via pericytes also occurs in the maintenance and optimal function of the tight junctions, with the entire molecular pathway still to be entirely explained.

Finally, a recent research direction is focusing on the stem cell properties of the pericytes. Several studies have demonstrated the ability of pericytes to differentiate in angioblasts, vascular cells, and even microglial cells in both in vivo and in vitro conditions [47]. Understanding pericytes’ differentiation mechanisms may help for future therapeutic approaches, as brain pericytes are actively involved in many pathologies, including neuroinflammation, stroke, and AD [48].

### 2.3. Astrocytes

The astrocyte is the most abundant glial cell in the CNS and is actively involved as a supportive factor in many neuronal processes, including the stability of the BBB. Because of their strategic localization between the neurons and the other components of the BBB (pericytes and BMECs), astrocyte structural integrity and correct functioning is essential for maintaining a healthy BBB. On the one hand, the astrocyte’s endfeet have direct contact with the BMECs, modulating the expression of endothelial carriers (P-gp and MRPs), thus sustaining the highly selective permeability of the BBB. In vitro studies conducted on BBB models have demonstrated that astrocytes increase the transendothelial electrical resistance (TEER), an electrical marker that shows the barrier-like properties of the BBB [49]. The astrocyte-BMEC crosstalk is bidirectional, with research highlighting the influence of endothelial-derived factors on astrocyte differentiation and growth [50]. On the other hand, astrocytes closely connect to the neurons and release important growth factors for neuronal survival. Similar to pericytes, astrocytes also produce Ang1 and TGF-β, but also specific growth factors such as the glial-derived neurotrophic factor (GDNF), which supports and repairs the surrounding neural structures [51].

An important aspect related to the astrocytes which are part of the BBB is their role in the osmotic equilibrium. At the astrocyte’s endfeet, specialized molecules such as aquaporin-4 (AQP-4) are the key players in regulating the water and ionic balance at the CNS level [52]. This has also diagnostic and therapeutic consequences: the detection of antibodies to AQP-4 is a highly suggestive marker for the autoimmune disorder neuromyelitis optica [53], while AQP-4 is recently considered a valuable target in treating cerebral edema [54]. There are still many other undiscovered subcellular structures (endfeet channels and transporters) involved in the bidirectional change between blood and brain parenchyma, with the astrocyte still having a lot to offer for future research.

### 2.4. Neurons

Neurons, the most specialized cells in the nervous system, are considered part of the broader concept of “NVU” rather than a component of the classical BBB. They are situated in the proximity of the BBB and have a significant rapport with the other cellular components of the barrier, most important with the astrocytes [55]. It is considered that virtually every neuron has its capillary or is situated at a maximum distance of 20 μm from a blood vessel [56]. The neuron–BBB interaction has bidirectional implications. On the one hand, BBB’s integrity is crucial in ensuring the physiological functions of the neurons, one of the most sensitive cells in the entire human body. The close connection to the BBB is facilitated by the astrocyte which acts as a mediator and assures a protective and supportive role for the neuron [57]. On the other hand, the changes in the brain milieu affect neuronal metabolism and behavior, with direct implications for the BMECs. Thus, neurons regulate the blood flow and the permeability of the cerebral microvessels through TJ and extracellular matrix modulation, along with other incompletely explained mechanisms [58].

Neurons’ close interaction with the other BBB components is also of great interest in different CNS pathologies, where concomitant neuronal and BBB dysfunctions are encountered. During ischemic stroke, the interruption of blood and oxygen supply leads to irreversible neuronal changes (including neuronal death), but also to BBB disruptions. The decreased expression of proteins forming the TJ, pericyte loss, and pathological activation of astrocytes are only some of the possible BBB alterations caused by the decreased blood supply and sustained by the damaged neurons [59]. Amyotrophic lateral sclerosis (ALS) is another neurological disorder relevant for the neuron–BBB crosstalk. Classically considered to be the result of the degeneration of motor neurons, recent studies also demonstrated BBB abnormalities in the brain of ALS patients, with astrocytic downregulation [60] and impaired endothelial repair being the most frequently described [61]. The degeneration of neurons located in specific brain areas is found also in the early stages of AD, concomitant with the upregulation of receptors for advanced glycation endproducts (RAGE) and downregulation of low-density lipoprotein receptor-related protein 1 (LRP-1) [62]. The exact mechanisms that explain the bidirectional crosstalk between neurons and the other components of the BBB and their alterations in various CNS disorders remains to be elucidated.

### 2.5. Oligodendrocytes, Microglia, and Other Cellular and Non-Cellular Components

Oligodendrocytes and microglia, not components of the BBB per se, are heavily involved in the physiological and pathological neurological conditions associated with the BBB. Initially considered to ensure insulation for the neuronal axon, oligodendrocytes seem to modulate the BBB tightness via several proposed mechanisms. Via TGF-β signaling, oligodendrocyte progenitor cells (OPCs) upregulate TJ proteins [63]. The Wnt/β-catenin signaling pathway, a highly conserved pathway that regulates fundamental cellular functions, is a key regulator activated by OPCs with an influence on the claudin-5 expression in the BMECs [64]. Moreover, the intercellular crosstalk between oligodendrocytes, OPCs, and BMECs is more complex than initially considered. Several studies showed that OPCs and oligodendrocytes enhance the integrity of the BBB by lowering its permeability [19]. Several pathways are discussed, such as the PDGF-BB/PDGFRα pathway [65], but the limited research on this topic suggests the implication of several other possible mechanisms.

Microglia, considered the equivalent of the immune cells at the CNS level, plays major roles in pathological conditions and is intensely related to neuroinflammation [66]. While the effects of microglia on the BBB are negligible in physiological conditions, microglia is one of the main actors that leads to BBB damage in many neurological disorders (multiple sclerosis, AD, and Parkinson’s disease) [67]. When turning into the M1 (pro-inflammatory) phenotype, microglia induces and/or sustains the chronic inflammatory state characterized by increased BBB permeability. Several hypotheses try to explain the M1 microglial activation and the subsequent microglia–BBB interactions that occur in pathological conditions. It is thought that factors resulting from BBB’s cellular components (BMECs, astrocytes, and pericytes) destruction favor, together with inflammatory markers, the activation of the microglia [68]. Subsequently, via pathological feed-forward loop mechanisms, microglial cells promote BBB alterations. This is of great importance also when recreating pathological conditions in BBB models in vitro, as detailed below in Section 4.

Finally, non-cellular BBB components must be also considered, with the basement membrane (BM) of capillary cells and the extracellular matrix (ECM) being two structures that show increasing interest among researchers. The BM, consisting of the vascular and the parenchymal parts, is a complex system that contains more than 30 types of proteins, with contractin-1, laminin, agrin, and perlecan among the most abundant ones [69]. A detailed understanding of the BM is of great importance for ensuring an accurate in vitro reproduction of the BBB, but also for the study of BBB changes (potential structural biomarkers) in pathological conditions. Alterations in the ECM have a similar impact on the BBB permeability as disruptions of the cellular components favor increased leukocyte and macromolecular trafficking through a normally impermeable barrier [70].

## 3. In Vitro Models of the Human Blood–Brain Barrier

The obvious structural complexity of the NVU raises big concerns when conducting studies in vitro, as the accurate replication of human biology is almost impossible. This was highly noticeable in earlier mono- and bicellular models, characterized by oversimplicity and a lack of similitude compared to the human in vivo BBB. In this context, the latest in vitro models are based on complex cellular co-cultures in their attempt to become more precise in mimicking the physiological BBB. This section reviews the evolution of BBB in vitro modeling, starting with the earlier animal-based monocultures and presenting the latest trends and technologies in the field, such as organ-on-a-chip (OOAC) and organoids that utilize inducible pluripotent stem cells (iPSCs). Table 1 summarizes the most relevant models utilized in the past and present, highlighting their main characteristics, and offering a rich list of references. Subsequently, Table 2 presents the advantages and limitations of each model in detail.

### 3.1. Transwell-Based Cellular Cultures

Historically, static monolayer BBB models were the first employed, despite their evident limitations. This was one of the most used models, as it was an easy approach based on the culture of endothelial cells from various origins (mouse, rat, porcine, and human) alone in a Transwell. In addition to its simplicity, researchers preferred this model as the quantification of the barrier integrity (the desired factor in drug penetrability studies) could be performed rapidly [104]. Besides measuring TEER, the direct observation of apical and luminal molecules which act as transporters or as markers for the BBB integrity is an advantage for in vitro studies [105]. However, because of the use of only one cell type, the BBB’s multiple properties could not have been correctly reproduced. The main limitation resides in the absence of the intercellular crosstalk between BMECs and the other BBB components which are now clearly demonstrated to play a huge role in modulating the BBB’s characteristics [46]. In addition, as with all static models, the lack of dynamic fluid flow and subsequently the absence of vascular shear stress make this in vitro model a far-from-reality reproduction, inadequate for studying complex neuropathological processes such as neurodegeneration.

Improvements were made, with the Transwell system being effortlessly adaptable to the co-culture of multiple BBB cells. Indeed, several approaches are still available and broadly used [89], one reason being the utilization of human-immortalized cells for simulating the in vivo BBB. Regarding the distance between BMECs and the other cellular components cultured in the Transwell system, two different approaches are available: the non-contact and the contact co-culture [106]. In both models, the endothelial cells are cultivated on the luminal side of the apparatus, while astrocytes, pericytes, and neurons can be grown at the abluminal side (contact) or at the bottom of the well (non-contact). There are fundamental differences between these two dispositions related to the modalities of cell-cell interaction, with relevant consequences depending on the research design. When thinking of possible co-cultures, several combinations are already validated: BMECs of murine, bovine, porcine, and human origin, modulated via intercellular connection with either astrocytes or pericytes. Triple cultures are also possible [84], with BMECs making up the constant cell line in combination with astrocytes and pericytes, or astrocytes and neurons. Finally, the most complex 2D co-culture is represented by the quadruple culture model, which encompasses all the cellular elements of the BBB [90].

### 3.2. BBB-on-a-Chip Technology

The precursors of the microfluidic brain chips are represented by the heterogeneous group of dynamic in vitro (DIV) models. DIV systems offer the possibility to grow a 3D cell culture in capillary-like support based on hollow fibers [91]. The principle is similar to the contact Transwell cultivation, with BMECs covering the inside of the lumen, while the other cellular components of the NVU are seeded extraluminally. The main advantage of DIV models over static co-cultures is the possibility to induce quasi-physiological shear stress, considered essential for BMECs phenotype modulation [92]. Other pros include the higher TEER, a relevant parameter when studying molecular/drug passage via the BBB [107], together with the ability to examine BBB alteration in hypoperfusion and reperfusion conditions [108]. On the other hand, the high costs along with the need for extensive skills to prepare and maintain the setup have limited the research based on DIV models [109]. Lastly, the use of hollow fibers with thick side walls imposes a huge limitation on the direct visualization of the cells and subcellular structures, with advanced and expensive microscopic techniques being almost mandatory.

The optimization of microdevices opened the way for a new subfield where engineering has brought enormous advances with a direct impact on BBB cell culturing. Organ-on-a-chip, including BBB-on-a-chip, represents a novel class of microfluidic devices that realistically simulates the dimensions and geometry of the human in vivo BBB [110]. The main advantage of this approach is the rapid and relatively low production costs, ensuring a precise 3D model along with the replication of pseudo-physiological shear stress [111]. The microfluidic channels also offer the possibility for migration and metastatic processes studies [109]. Some drawbacks must also be noted, with the lack of standardized parameters and characterization methods being the main limitation in the large-scale deployment of these devices.

New technology is however extremely helpful in perfecting the currently existing equipment. Three-dimensional printing, a technology that has an increasing demand in multiple domains, is becoming of interest in the microchip production field. While traditional OOAC fabrication was a potentially high-cost industry, involving time-consuming and complex multi-step lithographic processes, 3D printing offers a cheaper and faster alternative together with a simplification of the multi-step production. The digital technology allows a reduced operating time and minimal errors, even for complex shapes [112]. Material choice is an essential factor for the final product quality, as the wide range of possibilities (from thermoplastic to biocompatible polymers and resins) provides the chance to individualize BBB-on-a-chip devices according to research needs [113]. In this context, different printing techniques are available.

### 3.3. Organoids, iPSCs, and Other Future Directions

Despite their clear advantages related to the properties of mimicking the in vivo environment, microfluidic devices require specialized equipment and extensive skills; thus, their use in the research of the BBB is limited. The use of cell cultures is preferred, including 3D cultures such as multiple cells with BBB organoids (known also as spheroids) because of their greater benefits compared to classical ones [114]. A key feature of organoids is their ability to bring together different types of cells in close contact. As already demonstrated under in vivo conditions, the intercellular crosstalk among the cellular components of the BBB is mandatory for the cell’s phenotype determination and to ensure barrier characteristics [46,50]. Higher throughput combined with small size and great reproducibility are other benefits of organoids, especially with currently available detailed protocols [115]. Moreover, the greater accessibility to human iPSCs has opened the pathway for a more accurate and physiologically friendly in vitro BBB modeling. The latest research on spheroid models has surpassed the possibilities offered by the classical approaches, with new organoids containing up to six different cell types emulating in a highly accurate manner the in vivo human BBB [101]. BBB spheroids are a great tool when conducting studies on drug delivery according to many researchers [116], although TEER measurement is difficult and lacks a standardized measuring protocol.

Summing up the past and presently available techniques for in vitro BBB modeling, some shortcomings are immediately visible. No technique is currently able to embody all human BBB characteristics, with the most developed methods such as microfluidics and organoids necessitating extensive know-how and increased costs. Table 3 summarizes the most important characteristics of an ideal and modern in vitro model of the human BBB. In this context, the development of new, cheaper, and readily accessible technologies is mandatory to increase product availability and ensure a large-scale deployment for BBB research. Secondly, the creation of protocols regarding the generation and utilization of BBB spheroids is only in its infancy, and massive progress is mandatorily needed. Finally, embedding in vivo conditions to in vitro models, besides shear vascular stress, is another relevant aspect when thinking of the fragile hemostatic equilibrium of the human BBB that is extremely difficult to be reproduced in artificial conditions.

## 4. Designing the Pathological—In Vitro BBB Models for Alzheimer’s Disease

Besides its crucial role in physiological conditions, the BBB plays a major function also in pathological circumstances. AD is a relevant example of a frequent neurological disorder associated with important BBB damage [117]. Currently, no clear conclusion can be drawn whether the BBB alterations are the precursory factor for neurodegeneration or just a by-standing result of dementia. Thus, advanced in vitro BBB models may act as very helpful instruments for research on this highly discussed topic.

The first challenge when modeling the BBB in neurological conditions is the accurate recreation of the pathological milieu. Regarding AD, the lack of precise knowledge on the disease etiopathogenesis imposes the usage of several techniques that recreate the imbalances encountered in AD patients. As depicted in Table 4, a good BBB in vitro model for AD must mandatorily include the structural alterations determined by the pathological Aβ accumulation in the cerebral parenchyma [118]. Although not completely understood, the most significant BBB alterations in the AD brain include endothelial cells’ modifications such as increased pinocytosis, a decrease in mitochondrial content, and loss of impermeability in the TJs, along with the atrophy of pericytes and astrocyte endfeet swelling [119]. Non-cellular modifications should also be taken into consideration, with the accumulation of collagen and laminin in the basal membrane, a loss of actin expression in the vascular smooth cells, and the upregulation of AQP-4 as the main alteration with considerable impact on the permeability of the BBB [120]. The misfolded protein theory is not restricted to Aβ, other proteins such as hyperphosphorylated Tau protein (p-Tau) and alpha-synuclein are involved in the pathogenesis of AD and other neurodegenerative disorders [121].

**Figure 2:**
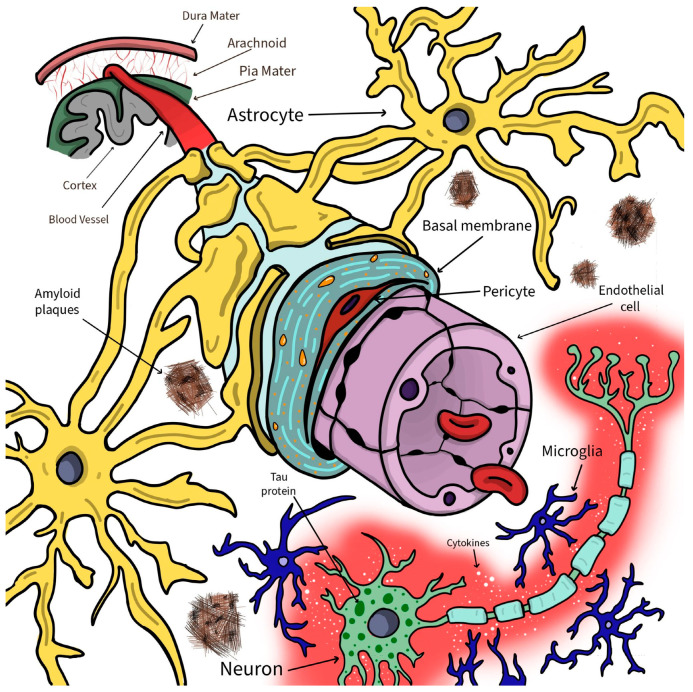
The most relevant pathophysiological changes of the neurovascular unit in AD. Many of the homeostatic processes of the BBB are impaired in Alzheimer’s disease. Vascular integrity is impaired by damage to the endothelial cells (purple), which lose their impermeability in the TJs, along with atrophy of pericytes (red), astrocyte endfeet swelling (yellow), and collagen and laminin accumulation in the basal membrane (blue). Amyloid-β (Aβ) builds up and organizes in plaques that surround the astrocytes and neurons. This causes neuroinflammation with the secretion of inflammatory cells and cytokines, with the central role played by microglia (dark blue). Within neurons (green), tau protein accumulates in neurofibrillary tangles (NFTs), which are associated with the accumulation of glial cells and neuronal dysfunction. (Magda Pîrțac designed this figure by using Adobe Fresco).

Additionally, other processes that are part of normal brain physiology get dysregulated, promoting BBB alterations and sustaining continuous neural degeneration. Neuroinflammation, including the myriad of cytokine- and immune-cell-related pathways, switches from neuroprotection to the promotion of CNS damage, inducing and maintaining BBB transformations [122]. In the context of neuroinflammation, the central role is played by the activated (M1 phenotype) microglia [123]. Similarly, oxidative stress via reactive oxygen species (ROS) and impaired perfusion influence supplementary BBB’s integrity, reducing its general selectivity and barrier properties [124]. All the abovementioned pathological changes encountered in AD which directly influence BBB’s structure are illustrated in Figure 2.

One straightforward way to simulate BBB alterations similar to the ones found in AD patients’ brains is to use neural progenitor cells (NPCs) that express characteristic mutations for the familial form of AD. The study conducted by Shin et al. in 2019 showed that NPCs expressing mutations in APP and APP/PSEN1 genes modulate the BBB characteristics in a predictive manner, increasing its permeability [120]. The main reason for BBB’s enhanced permeability was demonstrated to be a decrease in the expression of junctional proteins such as claudin-1, claudin-5, and VE-cadherin. Other cell types are also appropriate for AD in vitro modeling, with embryonic stem cells (ESCs) being a good example. These totipotent self-renewing cells, although forming teratomas when directly transplanted in vivo [125], are a great source for obtaining neural stem cells (NSCs), which subsequently differentiate into neurons, astrocytes, and oligodendrocytes [126]. A major advantage when using ESCs, NSCs, or IPSCs, similar to NPCs, is the possibility of inducing genetic mutations encountered in the familial form of AD, such as presenilin-1 (*PSEN1*) and *PSEN2* [127]. Similarly, the use of neuroblastoma cells may be a good choice when studying the cholinergic hypothesis of AD. As undifferentiated cells, they depict the immature cholinergic neuronal cells; however, as tumor cells, the toleration for oxidative stress and high glycolysis may lead to incorrect results in experiments [128].

Another way to simulate AD in artificial conditions is to induce specific pathological changes in initially healthy in vitro BBB models. This can be achieved via different mechanisms, with final results dependent on the used method. In this regard, the experiments conducted by Chen et al. [129] demonstrated the impact of human serum exposure on BBB’s integrity. The serum exposure determined an increase in Aβ and p-Tau levels, while single-cell transcriptomic analysis revealed reduced synaptic function in both neurons and astrocytes. Several other physical and chemical methods to increase BBB’s permeability were studied, especially in trials for CNS drug delivery. For example, incipient studies were based on the short-term opening of the BBB under the influence of osmotic shock triggers such as mannitol or arabinose [130]. More recently, alternative substances were tested, with encouraging results in animal models, but unconvincing results when translated into humans. Cereport, a selective bradykinin B_2_ receptor agonist which triggers vasodilatation, and Regadenoson, an A2A adenosine receptor agonist that disrupts TJs, transiently triggering the BBB opening, are worth mentioning [131].

A promising technique, and currently the only one which allows precise, transient, and noninvasive controlled BBB opening, is focused ultrasound (FUS) combined with microbubbles. The role of the microbubbles is to ensure a lower effective ultrasound intensity compared to the use of FUS alone, thus keeping the technique within safety margins [132]. Several mechanisms were proposed in order to explain the effect at the BBB level, the most relevant and already demonstrated being TJ disruption, cell membrane pore formation, and transcytosis enhancement [133]. BBB’s increased permeability secondary to FUS administration is a consequence of the downregulation in key protein expressions such as occludin, claudins, and ZO-1, and also relevant transporters, including P-gp [134]. One interesting topic related to FUS-induced alterations at the CNS level is related to the transient inflammatory effects. Neuroinflammatory reversible responses were detected with the help of positron emission tomography and were associated with astrocyte and microglia activation [135]. Low-intensity FUS is a powerful tool in opening the BBB via the abovementioned mechanisms and other incompletely explained structural and functional alterations. In order to assess whether FUS is also effective in modulating the BBB in vitro to recreate persistent neuroinflammation and/or neurodegeneration, future research is mandatorily needed.

## 5. Conclusions

The BBB, because of its uniqueness and complex structure, remains a huge challenge for in vivo studies; thus, accurate in vitro replication is essential for a better understanding of its behavior under physiological and pathological conditions. In neurodegenerative disorders, including AD, the BBB suffers multiple alterations that may be also relevant as early diagnostic markers and potential therapeutic targets. This is of huge importance, as currently conducted studies on AD possible therapies have unconvincing results.

In this context, significant improvements were made regarding in vitro modeling of the human BBB. A first step was to include several types of cells such as astrocytes and neurons along the BMECs in more complex cell culture models, including triculture and quadruple culture. Additionally, the use of human cells (in the form of immortalized human cell lines, human IPSCs, and human ESCs) offers a more authentic copy of the in vivo human BBB compared to earlier models based on rodent or porcine biological materials. Moreover, thanks to technological advancements, the broad dissemination of microfluidic devices and spheroids, and the development of new biologically friendly nanomaterials, limitations of past models can be overcome.

Although there have been numerous improvements and important gains of knowledge related to BBB modeling, there are still difficulties when trying to study AD on in vitro models mainly because of incomplete data on the disease. Neuroinflammation, the pathological activation of the microglia, oxidative stress, and the misfolded protein hypothesis are the most discussed theories at the present, and their simulation in BBB models under in vitro conditions represents a challenge for researchers over the next few years. Only by embodying multiple pathological conditions, the in vitro emulation of the human BBB will be an adequate replica of the in vivo counterpart. Finally, the continuous theoretical study of AD and the conduction of clinical trials on improved BBB models are mandatory directions used to achieve clinical and therapeutic results in the near future.

## Figures and Tables

**Table 1:** BBB in vitro models—main components and most relevant studies.

Proposed Model	Main Components [Reference(s)]
Static monolayer model	Mouse primary/immortalized BMECs [71] Rat primary/immortalized BMECs [72] Porcine primary BMECs [73,74] Human primary BMECs [75] Human immortalized BMECs [76] Human pluripotent stem cell-derived BMECs [77]
Co-culture in Transwell apparatus	Mouse primary BMECs + murine pericytes [78] Mouse primary BMECs + mouse/rat astrocytes [79] Rat primary BMECs + rat astrocytes [80] Immortalized human brain endothelial cells + astrocytes/pericytes [81] Primary human brain endothelial cells + astrocytes/pericytes [82]
Triculture in Transwell apparatus	Rat primary BMECs + rat astrocytes + rat pericytes [83] Porcine primary BMECs + rat/porcine astrocytes + rat/porcine pericytes [84] Immortalized human brain endothelial cells + astrocytes +pericytes [85,86,87,88]
Quadruple culture models	Human-induced pluripotent stem cells (hiPSCs) + multipotent fetal neural stem cells + astrocytes + pericytes [89,90]
Dynamic in vitro (DIV) models	Kirkstall QuasiVivo 600 (QV600)^®^ [91,92,93,94,95]
Microfluidic devices	Brain Chip (Chip-S1^®^, Emulate, Inc., Boston, MA, USA) [96,97,98,99,100]
Spheroids	Up to six different cell types (BMECs, astrocytes, pericytes, microglia cells, oligodendrocytes, and neurons) [101,102,103]

Abbreviations used in Table 1: BBB—blood–brain barrier; BMECs—brain microvascular endothelial cells; TEER—transendothelial electrical resistance.

**Table 2:** BBB in vitro models –advantages and limitations.

Proposed Model	Advantages	Limitations
Static monolayer model	Easy set-up protocol	Low TEER
	Reduced costs	Absence of the human in vivo BBB due to the lack of intercellular crosstalk with other types of cells (astrocytes, pericytes)
	Adequate for endothelial cells molecular studies	
Co-culture in Transwell apparatus	Cost-effective	Reduced intercellular contact (especially in non-contact co-culture models)
	Increased barrier stability Ability to study interactions between different cell types TEER closer to in vivo conditions	Tri- and quadruple culture: more difficult to grow compared to co-culture models
Dynamic in vitro (DIV) models	Higher TEER value	Exaggerated thickness of separating walls
	Ability to study the effects of flow cessation and reperfusion	Difficult visualization
	Ability to generate a quasi-physiologic shear stress	More difficult to set up compared to the Transwell-based models
		High costs
Microfluidic devices	3D model	Difficult set-up and maintenance
	Possibility to mimic the cerebral blood flow	Limited scalability
	Possibility to mimic shear stress (critical for BMECs phenotype) Adequate for studies on cell migration and metastasis	(Potential) high running costs Poor ability to quantify TEER (compared to Transwell co-cultures)
Spheroids	3D model Excellent cell contact Reduced de-differentiation	TEER measurement is very difficult (imprecise) Extensive skills required

Abbreviations used in Table 2: BBB—blood–brain barrier; DIV—dynamic in vitro; TEER—transendothelial electrical resistance.

**Table 3:** Requirements for an ideal (modern) in vitro model of the human BBB.

Technical Characteristic	Requirements for an Ideal In Vitro Model of the Human BBB
Production and set-up	Low-cost fabrication Easy set-up (no special training required) Improved reproducibility
Cells type and interaction	Use of human cells Co-culture (BMECs, astrocytes, pericytes, microglia cells, oligodendrocytes, and neurons) Low cell number required Increased intercellular contact
Physical properties	Mimic shear stress Mimic cerebral blood flow Dynamic model 3D structure High TEER value
Other parameters	High flexibility of the design Increased stability Precise control of the microenvironment conditions
Evaluation protocol	Easy visualization and inspection via microscopy Standardized quantification values Immediate measurements

Abbreviations used in Table 3: BBB—blood–brain barrier; BMECs—brain microvascular endothelial cells; TEER—transendothelial electrical resistance.

**Table 4:** Translating the pathophysiological AD hypotheses in accurate in vitro models of the BBB.

	Pathophysiological Changes
Most relevant pathophysiological AD hypotheses	Misfolded protein pathological accumulation (Aβ)
	Neuroinflammation
	M1 microglia activation
	Oxidative stress
	Gene–environment interactions
	Dysregulated autophagy
In vitro modeling of the BBB changes encountered in AD	Increased pinocytosis in BMECs
	Decreased mitochondrial content of BMECs
	Loss of TJ impermeability
	Atrophy of pericytes
	Swelling of astrocyte’s end feet
	Collagen and laminin accumulation in the basal membrane
	Upregulation of AQP-4

Abbreviations used in Table 4: AD—Alzheimer’s disease; AQP-4—aquaporin-4; BBB—blood–brain barrier; BMECs—brain microvascular endothelial cells.

## Data Availability

All data and materials supporting the results of the present study are available in the published article.
